# Efficacy of lifestyle interventions in physical health management of patients with severe mental illness

**DOI:** 10.1186/1744-859X-10-22

**Published:** 2011-09-19

**Authors:** Fernando Chacón, Fernando Mora, Alicia Gervás-Ríos, Inmaculada Gilaberte

**Affiliations:** 1 Clinical Research Department, Lilly SA, Madrid, Spain; 2 Servicio de Psiquiatría, Hospital Infanta Leonor, Madrid, Spain

## Abstract

Awareness of the importance of maintaining physical health for patients with severe mental illnesses has recently been on the increase. Although there are several elements contributing to poor physical health among these patients as compared with the general population, risk factors for cardiovascular disease such as smoking, diabetes mellitus, hypertension, dyslipidemia, metabolic syndrome, and obesity are of particular significance due to their relationship with mortality and morbidity. These patients present higher vulnerability to cardiovascular risk factors based on several issues, such as genetic predisposition to certain pathologies, poor eating habits and sedentary lifestyles, high proportions of smokers and drug abusers, less access to regular health care services, and potential adverse events during pharmacological treatment. Nevertheless, there is ample scientific evidence supporting the benefits of lifestyle interventions based on diet and exercise designed to minimize and reduce the negative impact of these risk factors on the physical health of patients with severe mental illnesses.

## Introduction

It is well known that patients with severe mental illnesses (SMIs) such as schizophrenia, depression, or bipolar disorder have worse physical health and reduced life expectancy compared to the general population [1-4]. There are data suggesting that patients with SMIs die on average between 13.5 and 32.2 years earlier than the general population. A recent study, using years of potential life lost (YPLL) as a measure of premature mortality showed that the mean YPLL in patients with SMIs was 14.5 compared with 10.3 for the general population [5]. Factors affecting patients with SMIs which contribute to these outcomes include more frequent physical comorbidities as compared to the general population [6], genetic predisposition to certain pathologies [7-9], eating habits and sedentary lifestyles [10,11], high levels of cigarette smoking and drug abuse [12-14], limited access to regular health care services [15,16], and potential adverse events arising during pharmacological treatment [17].

Weight gain and metabolism disturbances are among the well documented potential adverse events related to antipsychotic medication. A recently published meta-analysis shows that some second-generation antipsychotics (SGAs), such as olanzapine, lead to substantially more metabolic side effects than other SGAs [18]. The majority of studies used to perform the head-to-head comparisons with olanzapine were less than 1 year in length. Other studies have shown no statistical differences between olanzapine and other antipsychotics (typical and atypical) in weight gain and metabolic disturbances after 1 year of treatment [19-21], although significantly greater weight gain was found in olanzapine compared with risperidone and haloperidol after 3 months of treatment [22]. Regardless, a different pattern of weight gain in olanzapine compared with other antipsychotics is proposed [21].

In recent years the importance of physical health in patients with SMI has become increasingly recognized by the medical community [11] and, as a result, several guidelines and consensus recommendations [16,23-25] have been developed in order to define the standards for the management of physical health in this group of patients.

Several studies have investigated the genetic vulnerability of psychiatric patients with regard to physical health factors. Non-affective psychosis appears to be associated with reduced telomere content (a genetic marker of cellular senescence), elevated 2-h glucose levels, and increased pulse pressure, which are indices that have been linked to accelerated aging and a predisposition to diabetes mellitus and hypertension [26]. Additionally, one study has shown abnormal function of adult stem cells (SC) in these patients, suggesting a potential contribution to the high prevalence of medical problems in this population. However, these results have to be replicated and further examination of SC function should be conducted [27].

In addition to this genetic vulnerability, there are other risk factors that could be considered as modifiable. A recent position statement [28] has been published by the European Psychiatric Association (EPA), supported by the European Association for the Study of Diabetes (EASD) and the European Society of Cardiology (ESC), with the aim of improving the care of patients suffering from severe mental illnesses. Cardiovascular disease (CVD) is the most common cause of death in patients with SMI [2,29-32], and the statement proposes a series of interventions for the recommended management of CVD risk factors. Several of these risk factors are modifiable, including smoking, diabetes mellitus, hypertension, dyslipidemia, metabolic syndrome, and obesity [33].

Pharmacological approaches for the management of some CVD risk factors have been established [34-38], but the aim of this article is to review the role of lifestyle interventions that may contribute to the management of modifiable CVD risk factors in patients with SMI.

## Methods

The aim of this literature review was to highlight the efficacy of lifestyle interventions based on diet and exercise in the management of CVD risk factors in patients with SMI by evaluating a selective review of relevant literature focusing on the vulnerability of patients with SMI to these risk factors and the diseases associated. A Medline database literature search was performed for articles published between 2004 and 2010 using the term 'lifestyle intervention' linked with MeSH terms such as 'mental disorders', 'diabetes mellitus', 'hypertension', 'dyslipidemia', 'metabolic syndrome', 'obesity', and 'smoking cessation'. The reference sections of articles collected during the search were used to direct further inquiries. Cross-referencing of earlier reviews and original studies identified further information regarding the main topics of the search.

In all, 37 reports were retrieved during this search, 22 of which were original reports and 15 were reviews.

The impact of these kinds of interventions on obesity, diabetes mellitus, dyslipidemias, metabolic syndrome, hypertension, and smoking was evaluated. The prevalence and potential inter-relations of these CVD risk factors in patients with SMI were also evaluated, along with current evidence on how improvements in the management of the CVD risk factors may impact SMI patients' mortality and quality of life. Finally, the benefits of proactively implementing these lifestyle interventions will be discussed.

### Physical health vulnerability of patients with SMI

**Figure 1:**
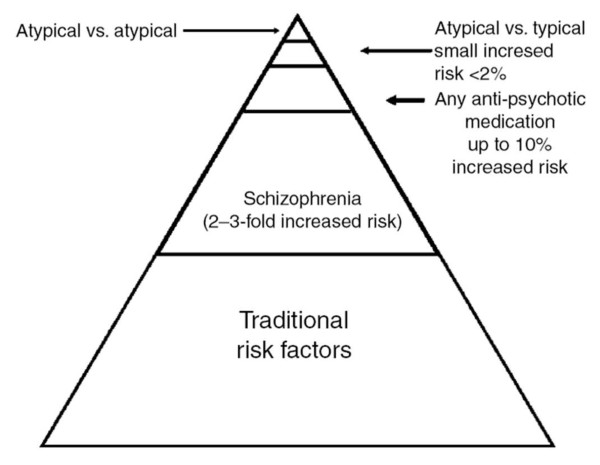
**Factors influencing the risk of diabetes mellitus among patients with schizophrenia**. Reprinted from Holt RI, *et al*. *Diabetes Obesity & Metababolism *2006, **8:**125-135. Reproduced with permission from John Wiley & Sons.

Although a strong genetic relationship between diabetes mellitus and schizophrenia has been established and specific loci have been observed that link schizophrenia and diabetes mellitus [8], the increased prevalence of diabetes mellitus in patients with schizophrenia [39] is fuelled by multiple factors. These factors include hereditary and environmental factors such as less healthy lifestyles and poorer health care, as well as side effects of antipsychotic medications. Nevertheless, much of the increased prevalence can be ascribed to traditional diabetic risk factors such as family history, physical inactivity, and poor diet (Figure 1) [40]. Therefore, any intervention focused on management of those factors will likely be successful in achieving a better control of diabetes mellitus.

Diabetes mellitus, like other CVD risk factors, approximately doubles the patient's risk of developing CVD [14]. The relationship between second-generation antipsychotics and glucose abnormalities is complicated due to the multifactorial mechanisms that underlie the development of diabetes mellitus [41], but it is widely accepted that the rate of diabetes mellitus is increased in people with schizophrenia in comparison with the general population [42]. Many other studies describe an increased prevalence as compared to the general population of diabetes mellitus in psychiatric patients [8,43], especially those with particular psychiatric illnesses such as schizophrenia or bipolar disorder, and this increase seems to be independent of age, race, gender, use of medication, or body mass [44]. People with schizophrenia are at an increased risk for the development of diabetes mellitus, with estimates suggesting prevalence between 15% and 20% [9]. The prevalence of diabetes mellitus in the bipolar disorder population may be as much as three times greater than in the general population [45].

Although there is not a consistent association between SMI and hypertension in the literature, a higher prevalence has been observed in patients with bipolar disorder and with anxiety disorders; this is not clear for schizophrenic patients [46]. In a meta-analysis comprising 12 papers on hypertension there was a pooled risk ratio of 1.11 (0.91 to 1.35), but there remains a weak association between SMI and hypertension [47].

Hypertension is highly important as a CVD risk factor [14] and, like other medical conditions, has a greater prevalence in patients with SMI [48]. However, a recent work shows that hypertension was the factor receiving more therapeutic care among the studied population; 69% of patients diagnosed with hypertension upon admission were receiving treatment [49].

Moreover, an unhealthy lifestyle related to diet habits and excessive sedentariness is an important contributor to CVD risk factors such as obesity, dyslipidemia, and metabolic syndrome. Worldwide obesity prevalence has a very wide range, from 80% in Nauru (an island nation in Micronesia in the South Pacific) to 9% in the Seychelles. The estimated prevalence in the Spanish adult population aged 25 to 60 years is 15.5% (13.2% in men and 17.5% in women) [50]. It is worth noting that in a study conducted in individuals with SMI in the community, 29% of men and almost 60% of women with SMI were obese [51].

**Figure 2:**
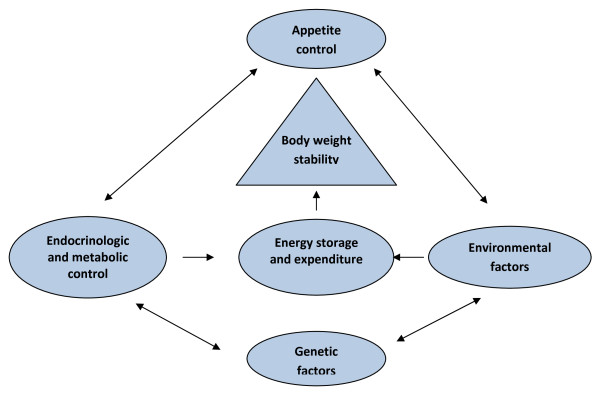
**Mechanisms of body weight regulation**. Adapted with permission from Wolterskluwer [56], Baptista *et al*. *CNS Drugs *2008, **22:**477-495.

The prevalence of obesity in patients with SMI is equal or higher than that of the general population [28,51-53], with antipsychotic medication as the contributing factor [52,54,55]. This effect has been observed to have different ranges for typical and atypical antipsychotics [56]. But medication is not the sole underlying factor for weight gain in patients with SMI, as there are multiple factors contributing to the risk of obesity among patients with schizophrenia including poor dietary habits, and inactivity [52]. Finally, it should be noted that body weight is regulated by a multifactorial mechanism composed of genetic and environmental factors, endocrinologic and metabolic control, and a delicate balance among energy intake, storage, and expenditure (Figure 2).

Obesity contributes to the risk of a number of diseases including diabetes mellitus, coronary artery disease, hypertension, stroke, gallbladder disease, osteoarthritis, and several kinds of cancers. All these factors can lead to further increases in morbidity and mortality [14,57,58].

The higher incidence of dyslipidemia in patients with SMI is unclear in the literature. Bresee *et al. *[59] found a slightly higher dyslipidemia rate in patients with schizophrenia compared with the no psychiatric population; this finding is in accordance with the high dyslipidemia rates (hypercholesterolemia (66%) and hypertriglyceridemia (26%)) found in other studies [49]. A meta-analysis including 11 papers on dyslipidemia [47] did not find an association between SMI and total cholesterol levels, but these studies in this meta-analysis were limited by their designs and so their conclusions must be considered carefully. The number of comparative studies of other lipids, such as high-density lipoprotein (HDL) cholesterol, was inadequate to conduct a meta-analysis. Lower HDL cholesterol levels in people with SMI found in two studies were not confirmed by any other studies.

The concept of metabolic syndrome has existed for many years and has several associated features such as central adiposity, hyperinsulinemia, hypertension, atherogenic dyslipidemia, decreased HDL cholesterol, elevated fasting triglycerides, and increased levels of prothrombotic proteins and inflammatory markers [60]. Metabolic syndrome prevalence varies according to several factors such as the diagnostic criteria [61] or country analyzed [61-63], but it is high in all analyzed studies; notably, a prevalence of 35% to 40% exists in the US population [62] and in developing countries studies have shown a wide range of prevalence, from 6.5% in India to 42.0% in Iran [63].

Prevalence of metabolic syndrome is higher in patients with SMI [7,60]; in the schizophrenic population the prevalence rate is 40% to 60% compared with 27% in the general population [42] and 40% in patients with bipolar disorder [64]. This increase is due partially to antipsychotic medications [7,65] and is associated with higher risk of CVD [60,65].

Other unhealthy lifestyle habits also increase the risk of CVD. It has been shown that there is a high proportion of smoking, alcohol abuse, and drug abuse in patients with SMI [12,13]; 85% of patients with SMI smoke, which is three times the rate found in the general population [14]. Smoking is considered as equivalent to metabolic syndrome in terms of CVD risk [28]. Approximately 60% of patients with depression and post-traumatic-stress disorder are smokers, while in patients with schizophrenia the prevalence of smoking can be as high as 65% to 90% [66]. Relative risk of smoking is elevated in patients with schizophrenia and bipolar disorder (elevated twofold to threefold in both illnesses) [28].

Smoking is a highly dangerous CVD risk factor for patients with SMI and raises the risk of CVD by 3; the risk of CVD is increased nearly 12-fold in individuals who have all risk factors compared with those who have none [14]. In the US, 40% of smoking-related deaths occur among mentally ill patients and substance abusers [67].

High-risk behaviors and unhealthy lifestyle habits are frequently found in patients with SMI, often as a result of social deprivation and occurring together with other factors such as more frequent physical comorbidities, genetic predisposition, limited access to regular health services, and potential adverse events arising from pharmacological treatment. These factors combine to contribute to this population's elevated risk for CVD. Choice of medication would seem to be as a modifiable risk factor. Any potential adverse effects of medication, particularly those that can contribute to increase the associated risk for physical illness should be balanced against their benefits in treating the mental illness, such as symptom control, improved quality of life, or reducing relapse, rehospitalizations or suicide rates.

Our review has focused in the modifiable factors associated to physical health and how lifestyle intervention strategies can modify the impact of such factors, especially those based on diet and exercise.

### Interventions and patients with SMI

Many examples in the literature examine how lifestyle interventions work in several aspects related to physical health [68,69]. Lifestyle interventions that facilitate the management of modifiable CVD risk factors are well established and, in most cases, have common characteristics, such as diet and exercise interventions [70,71]. This is a logical consequence of considering CVD risk factors as closely inter-related. Changes in a patient's lifestyle based on the successful incorporation of healthy eating and fitness habits can also reduce CVD risk factors (Table 1) [72]. We will see several examples of the efficacy of lifestyle intervention in every modifiable CVD risk factor separately and/or in combination.

**Table 1:** Therapeutic lifestyle changes for patients at high cardiovascular and metabolic risk: risk factors and goals/recommendations

Abdominal obesity	Physical inactivity	Atherogenic diet
7% to 10% loss of body weight from baseline	30 to 60 min of moderately intense aerobic activity daily	Saturated fat <7% of total calories

Caloric deficit of 500 to 1,000 kcal* daily		Reduce intake of *trans *fat

Physical activity		Dietary cholesterol <200 mg/dl*

		Total fat 25% to 35% of total calories

*To convert values to SI units: 1 kcal = 4.2 kJ; for cholesterol, 1 mg/dl = 0.02586 mmol/l.

Adapted with permission from American Journal of Medicine [72], Grundy SM. *Am J Med *2007, **120(Suppl 1):**S3-S8.

### Diabetes mellitus

Currently, many pharmacological approaches are available for reducing or delaying diabetes mellitus [35], but a key piece for the initial management of the disease for the majority of the affected population consists of lifestyle modification based on changes in dietary habits and physical activity [73].

Several studies have proven the efficacy of these lifestyle interventions in the management of diabetes mellitus in non-SMI patients as well [28,74]. In the early Malmö study [75], a lifestyle intervention based on diet and exercise facilitated normalized glucose tolerance in more than 50% of subjects with impaired glucose tolerance, and more than 50% of patients with diabetes mellitus were in remission after a mean follow-up of 6 years. In addition, improvement in glucose tolerance was correlated to weight reduction and increased fitness. The Diabetes Prevention Study (DPS) [76] showed that lifestyle intervention may prevent diabetes mellitus and reduce the risk of diabetes mellitus. This study showed that the reduction in the incidence of diabetes mellitus was directly associated with changes in lifestyle as well.

The efficacy of lifestyle interventions in patients with SMI has been demonstrated clearly. One study investigated a population of patients with schizophrenia to evaluate the efficacy of lifestyle interventions (based on psychoeducational, dietary, and exercise programs) and metformin, both alone and in combination, for antipsychotic-induced abnormalities in insulin sensitivity [77]. It showed that lifestyle intervention and metformin, both alone and in combination, can improve insulin sensitivity induced by antipsychotic medications. In addition, lifestyle intervention plus metformin was superior to lifestyle intervention plus placebo in decreasing insulin and Insulin Resistance Index (IRI), while metformin alone has the same effect on insulin sensitivity as lifestyle intervention plus metformin. Metformin was superior to lifestyle intervention plus placebo in decreasing fasting glucose, insulin levels, and IRI levels. All three intervention groups were found to have a significant advantage over placebo in improving weight gain and insulin sensitivity in patients with schizophrenia. The addition of a lifestyle intervention seems to be more efficacious than pharmacological treatment alone in the management of diabetes mellitus variables.

Due to the limited effect of pharmaceutical treatment for diabetes mellitus on glycemic control, lifestyle interventions designed to prevent an increase in blood glucose must be initiated as soon as possible. Ideally, such interventions should begin before the clinical symptoms of diabetes mellitus appear and before glucose levels are high enough to be classified in the range for diabetes mellitus. The risk of complications has already begun in the prediabetic phase before the patient's blood glucose levels reach diagnostic cut-off points for diabetes mellitus. In light of this, waiting until individuals attain the diagnostic criteria for diabetes mellitus will result in significant morbidity and mortality from cardiovascular disease [78].

### Hypertension

Lifestyle interventions have proven efficacy in the management of hypertension. The PREMIER trial [79] tested the effects of two multicomponent lifestyle interventions on patients with hypertension relative to a control group and observed reductions of 12% to 14% in estimated CVD risk (estimated from the Framingham risk equations). A review of lifestyle interventions with intentional weight loss showed that those lifestyle interventions were effective in reducing systolic blood pressure, although the evidence for diastolic blood pressure was less convincing [80]. A reduction in hypertension values was observed in patients with SMI who followed a lifestyle intervention based on diet and exercise, but that decrease was not statistically significant [81]. Lifestyle changes such as stopping smoking, reducing salt intake, reducing body weight, and increasing exercise may be sufficient to reduce mildly elevated blood pressure [28].

### Obesity

Programs of lifestyle intervention designed to establish good nutritional and exercise habits have showed efficacy in reducing weight gain and in the treatment of obesity. A systematic review performed to evaluate the effectiveness of long-term lifestyle interventions in preventing weight gain found a wide range of results in the different studies reviewed, but it was apparent that diet, alone and with the addition of exercise and/or behavioral therapy, led to significant weight loss and improvement in metabolic syndrome and diabetes mellitus for at least 2 years, compared with a control group that received no treatment [70].

These kinds of lifestyle interventions have proven efficacy in reducing weight gain in patients with SMI with very promising results [51,82,83], and preventive approaches have the potential to be more effective, acceptable, cost efficient, and beneficial [54]. A structured program sponsored by Eli Lilly and Co. (generally called Solutions for Wellness) is based primarily on exercise and diet counseling and has been performed in several countries. When carried out in a US population, this program demonstrated that people with mental illness have the desire to improve their health and well-being [84]. Patients achieved a mean body mass index (BMI) reduction of 0.93 kg/m^2 ^at the end of the 6-month observation period [85,86], with results similar to those of another study which showed differences in weight gain between the intervention group and the standard care group, the latter of which had gained a significant amount of weight by the end of the study [86].

A 4-week study carried out in an Irish population showed that by discontinuation of engagement with the program, only 14/47 (30%) patients had gained weight during a mean follow-up of 24 days (median 14 days) and the remainder either maintained their weight or lost weight [87]. Similar results in BMI reductions have been observed in a Korean population in a study of 12 weeks' duration [88,89]. These programs have also demonstrated efficacy in the population of patients with SMI in long-term weight management (2, 4, and even 8 years) [90,91].

Although these studies have shown the efficacy of Solutions for Wellness programs in the management of parameters such as weight gain, BMI, and abdominal circumference, the results are not really conclusive due to limitations in the studies design, such as the absence of a control group. It would be advisable to perform additional studies with more control and detailed designs to evaluate deeper the efficacy of this program.

### Dyslipidemia and metabolic syndrome

Effective management of dyslipidemia and metabolic syndrome may be implemented by working on the reduction of obesity and weight gain. A good example of a lifestyle intervention program with the objective of managing weight gain that has been induced by antipsychotics in patients with SMI is the study by Poulin *et al*. performed in a Canadian population [92]. It was a prospective, comparative, and open-label study carried out on a total of 110 patients with schizophrenia and schizoaffective or bipolar disorders being treated with atypical antipsychotics. Of these patients, 59 (experimental group) participated in an 18-month weight-control program that included dietary education and physical activity counseling as well as a structured, supervised, facility-based exercise program in a small gymnasium, consisting of 90 min of physical activity counseling provided at the beginning of the study and delivered by a nutritionist and a psychiatric nurse.

A kinesiologist supervised small groups who were devoted to exercise sessions performed for 60 min twice a week. The control group consisted of 51 patients who did not participate in the clinical program. Anthropometric and metabolic parameters were analyzed.

At the study endpoint, investigators observed reductions in the active group for the anthropometric variables that differed substantially from the control group: body weight (difference of 6.7 kg, *P *<0.01), BMI (difference of 3.2 kg/m^2^, *P *<0.01), and waist circumference (difference of 9.3 cm, *P *<0.01). Regarding metabolic parameters, at the study endpoint significant mean differences between the two groups were observed in total cholesterol, low-density lipoprotein (LDL) cholesterol, HDL cholesterol, triglycerides, and fasting glucose concentrations. Glycosylated hemoglobin (HbA1C) significantly decreased (-11.4%) compared to baseline in the active group. This study demonstrates that not only body weight but metabolic risk profile can be effectively managed with a weight-control program that includes physical activity.

Furthermore, it has been shown that relatively small weight loss can confer health benefits. A loss of just 5% of body weight in obese individuals may result in clinically meaningful reductions in morbidity and mortality, as well as additional improvements in glucose control in those with diabetes mellitus. Similarly, weight reduction in an overweight (BMI >25) individual may lead to reduction in blood pressure [93]. Even moderate weight loss (10% or less) has been associated with improved insulin action, decreased fasting blood glucose, and decreased need for diabetes mellitus-related medications [94].

An analysis of the efficacy of lifestyle intervention programs in the reduction of blood lipids in patients with SMI [94,95] versus a population with no mental illness [76,96] reveals a moderate effect that is only significant in the case of triglycerides; its efficacy with regard to LDL and HDL is less clear and the statistical significance varies among studies. Lifestyle interventions have demonstrated efficacy in reducing rates of metabolic syndrome [65,95], in which the key component for change is the reduction of body fat percentage. Weight loss is the major determinant in maximizing effectiveness in improving metabolic syndrome parameters [74]. Small changes in body fat can elicit changes in metabolic syndrome, which may ultimately translate into changes in risk of CVD [95].

### Smoking cessation

The effectiveness of lifestyle intervention in smoking cessation has been studied when the intervention consists only of lifestyle counseling and its combination with pharmacotherapy. The effectiveness of lifestyle interventions (including pharmacological treatment) in smoking cessation has been proven in patients with SMI [97-100]. However, there are data that suggest that patients with a history of mental health disorders are less likely to quit smoking and have lower cessation rates than the general population [66]. Lifestyle interventions concerning smoking cessation seem to be more effective when a pharmacological treatment (nicotine replacement therapy or bupropion) is adjuvant [98,99]. Rigotti *et al. *[101] performed a systematic review to study the effectiveness of smoking cessation interventions. The effectiveness of lifestyle interventions in smoking cessation consisting of counseling is established, and the addition of a pharmacological treatment increases the rate of quitting.

## Conclusions

The physical health of patients with SMI should be part of the field of action of psychiatric practitioners, and global health (physical and mental) is a universal goal at present time. The objective of reducing the risk of CVD in patients with SMI is crucial given the particular vulnerability of this population to physical illnesses and the fact that CVD is the most common cause of death in patients with SMI.

**Figure 3:**
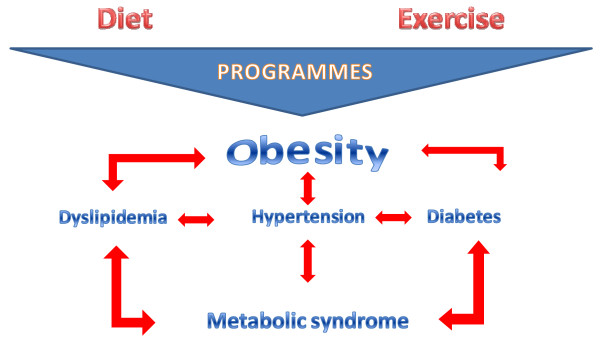
**Inter-relationship of cardiovascular disease (CVD) risk factors and action of lifestyle intervention programs**.

Strong evidence confirms the efficacy of lifestyle interventions based on diet and exercise in the management of CVD risk factors. The clear inter-relation and interdependence among all CVD risk factors means that improving one of them through lifestyle intervention programs can lead to a concomitant improvement in the other factors as well (Figure 3). This is particularly evident in the case of obesity or weight gain, where all lifestyle interventions based on diet and exercise that leads to weight reduction achieve benefits in other physical health parameters, such as metabolic ones.

It may seem obvious to conclude that a healthy lifestyle with healthy nutrition and regular physical activity is efficacious in achieving good physical health, even in patients with SMI. But we can only wonder about the number of patients with SMI presenting an increase in one of the CVD risk factors invited to participate in a lifestyle intervention program, notwithstanding the strong scientific evidence supporting their efficacy for the improvement of those factors. Moreover, if we take into account that several studies suggest that a genetic vulnerability exists in these patients independent of the antipsychotic treatment [8,26], the preventive implementation of lifestyle intervention programs should be considered good practice in treating these patients.

## Competing interests

FC, AG-R and IG are full-time employees of Lilly Spain. FM has served as paid spokesperson for Lilly Spain.

## Authors' contributions

FC: contributed to the review conception and design, carried out the selective review of the literature, carried out the analysis and interpretation of data, and drafted the manuscript. FM: contributed to the analysis and interpretation of data, and revised the manuscript critically for important intellectual content. AGR: contributed to the review conception and design, carried out the selective review of the literature and the analysis and interpretation of data. IG: responsible for the review conception and design, and revised the manuscript critically for important intellectual content, and gave final approval of the version to be published. All authors read and approved the final manuscript.
